# BRAF Mutation as a Potential Therapeutic Target for Checkpoint Inhibitors: A Comprehensive Analysis of Immune Microenvironment in BRAF Mutated Colon Cancer

**DOI:** 10.3389/fcell.2021.705060

**Published:** 2021-07-26

**Authors:** Shuyi Cen, Kun Liu, Yu Zheng, Jianzhen Shan, Chao Jing, Jiale Gao, Hongming Pan, Zhigang Bai, Zhen Liu

**Affiliations:** ^1^Department of Oncology, Sir Run Run Shaw Hospital, Zhejiang University School of Medicine, Hangzhou, China; ^2^Department of General Surgery, Beijing Friendship Hospital, Capital Medical University, Beijing, China; ^3^Clinical Center for Colorectal Cancerm, Capital Medical University, Beijing, China; ^4^Beijing Key Laboratory of Cancer Invasion and Metastasis Research, Beijing, China; ^5^National Clinical Research Center for Digestive Diseases, Beijing, China; ^6^Department of Oncology, The First Affiliated Hospital, Zhejiang University School of Medicine, Hangzhou, China

**Keywords:** BRAF, colon cancer, immune microenvironment, immunotherapy, therapeutic target

## Abstract

BRAF mutated colon cancer presents with poor survival, and the treatment strategies are controversial. The tumor microenvironment, which plays a key role in tumorigenesis as well as responses to treatments, of this subtype is largely unknown. In the present study, we analyzed the differences of immune microenvironments between BRAF mutated and BRAF wild-type colon cancer utilizing datasets from The Cancer Genome Atlas and Gene Expression Omnibus and confirmed the findings by tissue specimens of patients. We found that BRAF mutated colon cancer had more stromal cells, more immune cell infiltration, and lower tumor purity. Many immunotherapeutic targets, including PD-1, PD-L1, CTLA-4, LAG-3, and TIM-3, were highly expressed in BRAF mutated patients. BRAF mutation was also correlated with higher proportions of neutrophils and macrophages M1, and lower proportions of plasma cells, dendritic cells resting, and T cells CD4 naïve. In conclusion, our study demonstrates a different pattern of the immune microenvironment in BRAF mutated colon cancer and provides insights into the future use of checkpoint inhibitors in this subgroup of patients.

## Introduction

Colon cancer is one of the leading causes of cancer-related deaths worldwide. Colon cancer accounts for 6.0% of new cancer cases, with more than a million new cases of colon cancer identified in year 2020, and 5.8% of cancer deaths (Sung et al., 2021). Both environmental and genetic factors, such as mutated genes, inflammation, radiation, and hereditary disorders, contribute to tumorigenesis of colon cancer (Chan and Giovannucci, 2010).

The BRAF gene is one of the key genes in the RAS–RAF–MEK–ERK–MAP kinase pathway, which regulates many fundamental cellular processes such as cellular growth, proliferation, differentiation, migration, and apoptosis (Dhillon et al., 2007). There are several subtypes of BRAF mutations, and the most common one lies at codon 600 (BRAF V600E), accounting for about 95% BRAF mutated cases (Molina-Cerrillo et al., 2020). The BRAF mutation accounts for around 10% cases of colon cancer and serves as a strong negative prognostic marker for patients (Davies et al., 2002; Gonsalves et al., 2014; Barras et al., 2017). Mutated BRAF tumors are prone to have elevated kinase activity and promoted cell proliferation through MAPK cascade, and therefore subject to a different pathological and immunological patterns (Davies et al., 2002). BRAF mutation is often associated with high microsatellite instability (MSI) and is frequently linked to proficient mismatched repair bases in DNA (Rajagopalan et al., 2002). BRAF mutated colon cancer often derives from sessile serrated adenoma and tends to be high grade, poorly differentiated, and located on right-sided colon, and has more mucinous component, more peritoneal and lymph node metastases, but less lung metastases (Matos and Jordan, 2018; Lee et al., 2019).

Tumor microenvironment plays an important role in tumorigenesis and cell proliferation. Aberrant infiltrated immune cells in human tumors not only fail to restrain tumor growth but also promote tumor escape from the host (Whiteside, 2008). Dysregulated molecular and cellular pathways in the tumor microenvironment also contribute to inhibition of apoptosis. T cells, natural killer (NK) cells, lymphocytes, fibroblasts, and many other immune cells have been proven to play important roles in tumor development in colon cancer patients (Kather and Halama, 2019). Recent studies demonstrated that immune microenvironment in BRAF mutated colon tumors might lead to resistance to conventional therapies through regulation of composition of immune cell infiltration and chemokines (Reddy et al., 2016; Croce et al., 2019). Mitogen-activated protein kinase (MAPK) inhibition gives rise to suppression of immunosuppressive factors such as interleukin-10 (IL-10), vascular endothelial growth factor (VEGF), programmed cell death protein 1 (PD-1), regulatory T cells, etc., and targeting BRAF mutation was associated with lower expression of these immunosuppressive factors (Reddy et al., 2016; Chen and Hurwitz, 2018; Yuan et al., 2018). However, despite the importance of the immune microenvironment in the development and treatment of BRAF mutated colon cancer, few studies have yet investigated the pattern of tumor microenvironment in BRAF mutated colon tumors. In the present study, we pooled The Cancer Genome Atlas (TCGA) and Gene Expression Omnibus (GEO) datasets to explore the immune landscape of BRAF mutated colon cancer and to validate the important immune markers in patient specimens.

## Materials and Methods

### Gene Expression Datasets

Gene expression profiles of colon cancer were downloaded from the TCGA portal^1^ and the GEO database (accession number: GSE39582) directly. Clinical information of patients were also obtained from the TCGA portal and GEO database, including gender, age at diagnosis, stage, mutation status, survival time, tumor location, as well as MSI status. Immune scores, stromal scores, ESTIMATE scores, and tumor purity were calculated using the ESTIMATE (Estimation of STromal and Immune cells in MAlignant Tumour tissues using Expression data) algorithm, which utilizes gene expression signatures of tumor cells to infer the fraction of stromal and immune cells in tumor samples (Yoshihara et al., 2013). This calculation was performed using estimate package in R software.

### Identification of Differentially Expressed Genes (DEGs)

Data analysis was performed using R package limma (Ritchie et al., 2015). Fold change > 2 and multiple-testing adjusted *p*-value < 0.05 were set as the cutoffs to identify DEGs in both the TCGA and GEO cohorts.

### Functional Analysis and Heatmaps

The Kyoto Encyclopedia of Genes and Genomes (KEGG) analysis was employed to understand the potential function and pathway enrichment of DEGs using the clusterProfiler package in R software (Yu et al., 2012). The adjusted *p*-value < 0.05 was considered statistically significant. Heatmaps were generated using TBtools^2^ (Chen et al., 2018).

### Survival Analysis

Log-rank test for Kaplan–Meier curve was applied to illustrate the association between overall survival of patients and gene mutation status using survival^3^ and survminer^4^ package in R software. A value of *p* < 0.05 was considered as statistically significant.

### Overall Survival-Related Genes

The overall survival related genes were generated using the TIMER website, which is an online tool for analyzing genes and tumor-infiltrating immune cells in TCGA portal^5^ (Li et al., 2017, 2020).

### Immune Cell Analysis

Fractions of infiltrating immune cells of colon cancer patients were analyzed using Cibersort, an online tool to provide the estimation of the fraction of many immune cells using gene expression profiles^6^ (Newman et al., 2015).

### Tissue Immunohistochemistry (IHC)

Tissue samples were collected from 43 colon cancer patients receiving treatments at Beijing Friendship Hospital, Capital Medical University. The mutation status was determined by next-generation sequencing. All stainings used 4-μm-thick formalin-fixed paraffin-embedded tissue sections. The slides were baked, deparaffinized, rehydrated, washed, and then added with 0.3% hydrogen peroxide at room temperature to block the activity of endogenous peroxidase. For antigen retrieval, the sections were boiled for 25 min in citric acid-based buffer at pH 6.0 and EDTA-based buffer at pH 8.0, cooled to room temperature, and rinsed with PBS three times for 3 min. Slides were then incubated with primary antibodies, which were diluted using 1% BSA at 37°C for 1 h. After being rinsed, the sections were incubated in secondary antibodies (Maxim, Fuzhou, China) at room temperature for 15 min and stained with DAB detection kit (Gene Tech, Shanghai, China). Each section was washed as before and observed at ×200 magnification using a light microscope. Primary antibodies used included PD-1 (1:300, no. EM1707-60), PD-L1 (1:100, no. ET1701-41), TIM-3 (1:100, no. EM1701-19), BST2 (1:100, no. ET1706-46), CALB2 (1:100, no. ET1705-19), ENO2 (1:100, no. ET1610-96), Syndecan-1 (1:50, no. ET1703-42), HLA-DR (1:100, no. ET1702-51), Mye (1:100, no. ET1703-21) (all from Huabio, Hangzhou, China), and CTLA-4 (1:1,000, no. ab237712) and LAG-3 (1:1,000, no. ab180187) (both from Abcam, Cambridge, United Kingdom). For quantitative analysis, the staining scores were defined as 0 (negative), 1 (weak), 2 (moderate), and 3 (strong). Scores for the percentage of tumor cells for 0–10, 11–25, 26–50, 51–75, and >75% were classified as 0, 1, 2, 3, and 4, respectively. The scores of the staining intensity were multiplied by the scores of the percentage of stained cells.

### Statistical Analysis

Univariate analyses between gene mutation and clinical characteristics were compared using the log-rank test. The Student’s *t*-test was applied to analyze the association between gene mutation and stromal/immune/ESTIMATE scores and tumor purity. Log-rank test for Kaplan–Meier curves was conducted to assess the association between overall survival and gene mutations or expression levels. A value of *p* < 0.05 was considered statistically significant. The Venn diagram was plotted by R software.

## Results

### Clinical Characteristics of Patients From the TCGA and GEO Dataset

There was a total of 396 patients from the TCGA cohort with available BRAF mutation status, and 59 (14.9%) patients were BRAF mutated, as shown in Table 1. Patients carrying BRAF mutant tend to be female (*p* = 0.0029) and older (*p* = 0.0102) compared to BRAF wild-type patients. The tumors are also more likely to be at an advanced stage (*p* = 0.0226), more often located in the right colon (*p* < 0.0001), and prone to be MSI-H (*p* < 0.0001). Similar results were found in patients from the GEO cohort; BRAF mutated patients were more likely to be female (*p* = 0.0018), older (*p* < 0.0001), and had the tumor at the right colon (*p* < 0.0001).

**TABLE 1:** Patient characteristics in the TCGA and GEO dataset.

**Patient characteristics**		**TCGA**			**GEO**		
**BRAF status**		**Mut**	**WT**	***P*-value**	**Mut**	**WT**	***P*-value**
Total		59	337		51	461	
Gender	Female	39	150	0.0029	34	200	0.0018
	Male	20	185		17	261	
	NA		2				
Age	45	1	30	0.0102	0	35	<0.0001
	45–65	14	122		3	158	
	65+	44	183		48	267	
	NA		2			1	
Stage	Stage I	10	53	0.0226			
	Stage 2	32	118				
	Stage 3	14	95				
	Stage 4	3	57				
	NA		14				
T stage	T1	2	8	0.9609	2	8	0.4108
	T2	10	56		4	33	
	T3	40	225		29	299	
	T4	7	45		15	0.	
	NA		3		1	23	
Tumor location	right	49	176	<0.0001	44	164	<0.0001
	Left	4	145		7	297	
	NA	6	16		0	0	
MSI status	MSI-H	41	26	<0.0001			
	MSI-L	5	65				
	MSS	10	227				
	NA	3	19				

### Immune Score Analysis

**FIGURE 1:**
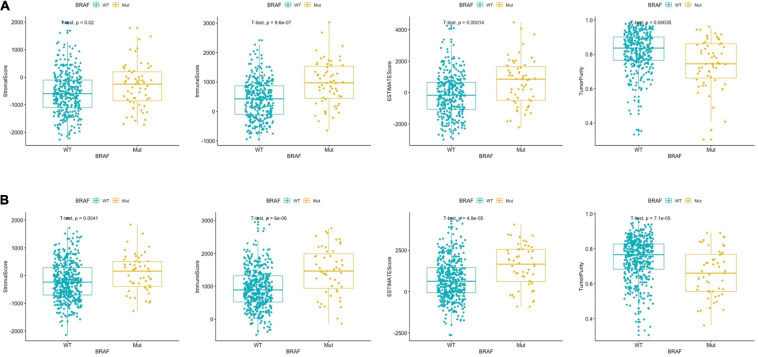
BRAF mutation in colon cancer is presented with significantly different stromal scores, immune scores, ESTIMATE scores, and tumor purity in the TCGA dataset **(A)** and GEO dataset **(B)**. Student’s *t*-tests were used for all analyses.

The ESTIMATE algorithm was established by Yoshihara et al. (2013) to predict the level of infiltrating stromal and immune cells. The levels of stromal cells and immune cells were defined as stromal score and immune score, respectively. These two scores form the basis for the ESTIMATE score to infer tumor purity. Stromal cells are one of the key players in tumor proliferation, invasion, and drug resistance, while infiltration of immune cells could serve as a prognostic indicator in cancer patients. We found that BRAF mutant colon cancer had higher stromal score (*p* = 0.02), immune score (*p* < 0.0001), ESTIMATE score (*p* = 0.0001), and lower tumor purity (*p* = 0.0003) (Figure 1A). The results suggest that BRAF mutant tumor tissues carry more stromal cells, more immune cell infiltration, and had lower tumor purity. Results from the GEO patients were in accordance with those of the TCGA patients. As shown in Figure 1B, BRAF mutated tumor had higher stromal score (*p* = 0.0041), immune score (*p* < 0.0001), ESTIMATE score (*p* < 0.0001), and lower tumor purity (*p* < 0.0001).

### Overall Survival Analysis

**FIGURE 2:**
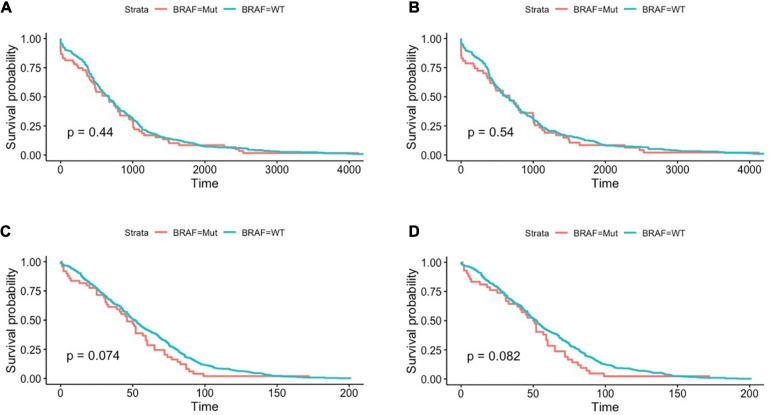
Patients with BRAF mutation had no significant differences in terms of overall survival in the TCGA dataset, as shown in all-stage patients **(A)** and stage III/IV patients **(B)**. Similar results were found in GEO datasets, as shown in all-stage patients **(C)** and stage III/IV patients **(D)**. Student’s *t*-tests were used for all analyses.

The literature has reported that BRAF mutated colon cancer patients had a much poorer survival rate that wild-type patients (Phipps et al., 2015; Matos and Jordan, 2018; Taieb et al., 2019), we therefore analyzed overall survival in the two cohorts. However, as shown in Figures 2A,C, there was no significant survival difference between BRAF mutated and wild-type patients. Further analysis in patients with T3 or T4 stage (Figures 2B,D) also revealed no difference in overall survival. This contradictory result may attribute to the fact that patients at these two cohorts were the ones who received radical surgery and were subjected to fewer metastasis. Brevity of patient survival information is another limitation of survival analysis in these two cohorts.

### DEGs and Enrichment Analysis

**FIGURE 3:**
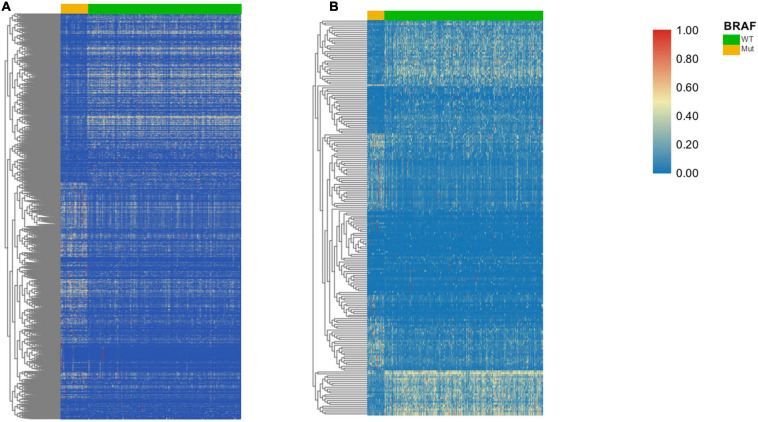
Heatmaps of DEGs in the TCGA **(A)** and GEO dataset **(B)**.

**FIGURE 4:**
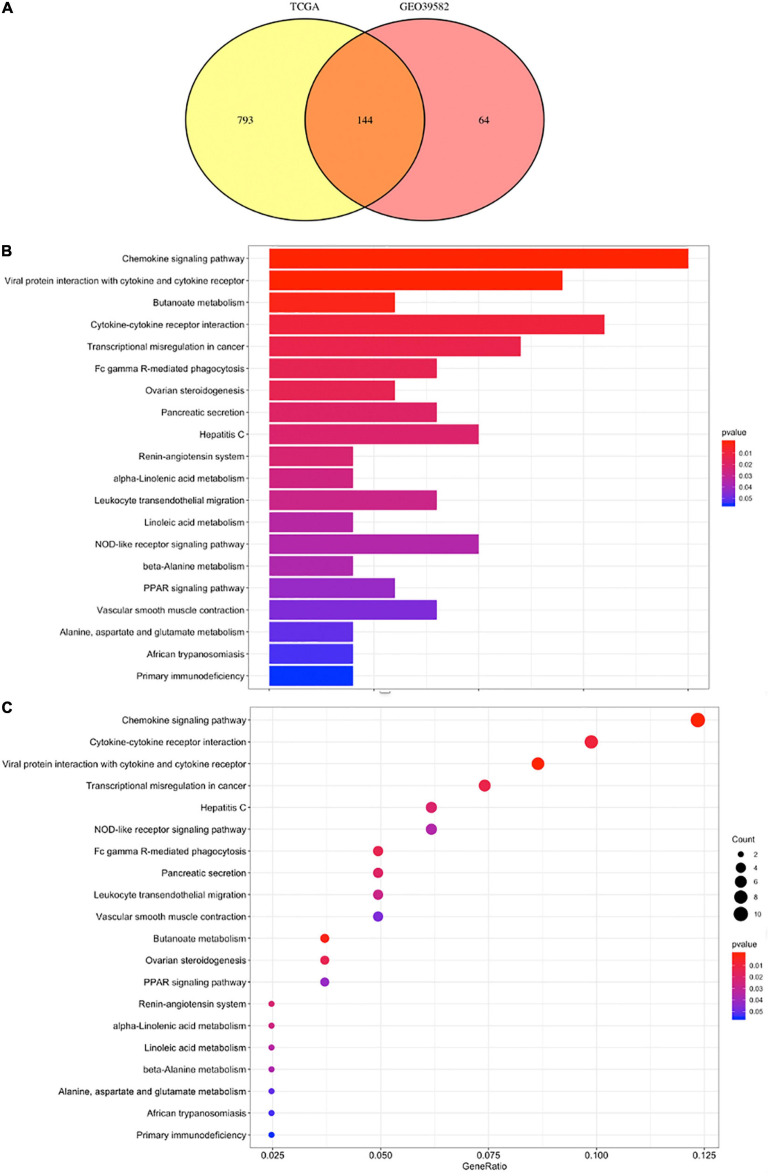
DEGs between BRAF wild-type and BRAF mutated patients **(A)** and their KEGG graphs **(B,C)**.

A total of 973 genes in the TCGA cohort and 208 genes in the GEO cohort were found to have significantly different expression levels between BRAF mutated and BRAF wild-type tumors, respectively. Heatmaps of these genes are shown in Figures 3A,B. Among them, 144 genes are differentially expressed in both the TCGA and GEO cohorts. These overlapping 144 genes were selected to perform KEGG pathway enrichment analysis (Figure 4A). Figures 4B,C suggest that chemokine signaling pathway, cytokine receptor interaction, viral protein interaction with cytokine and cytokine receptors, and transcriptional misregulation in cancer might be the leading pathways that bring about alteration in tumorigenesis in BRAF mutated colon cancer.

**FIGURE 5:**
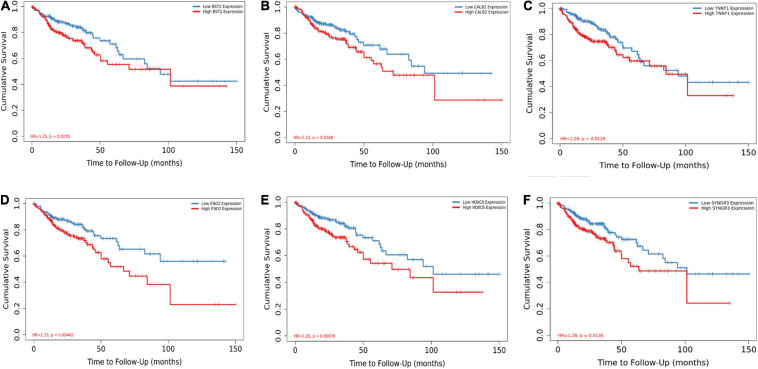
BST2 **(A)**, CALB2 **(B)**, TNNT1 **(C)**, ENO2 **(D)**, HOXC6 **(E)**, and SYNGR **(F)** are associated with overall survival. Log-rank test for Kaplan–Meier curves was conducted to assess the association between overall survival and expression levels.

**FIGURE 6:**
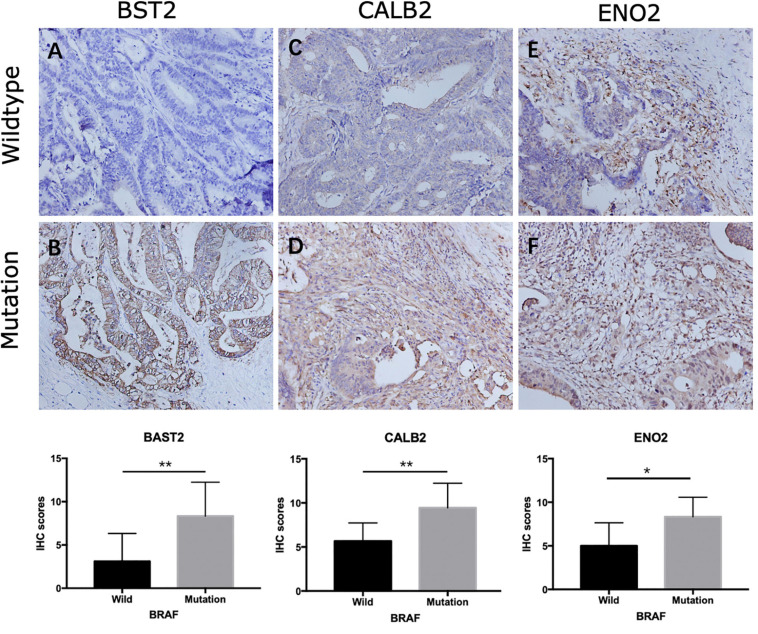
Representative IHC staining showing expression of overall survival-related genes in colon cancer. Staining of BST2 **(A,B)**, CALB2 **(C,D)**, and ENO2 **(E,F)** in BRAF wild-type **(A,C,E)** and BRAF mutated **(B,D,F)** samples at magnification ×200. *p < 0.05, **p < 0.01, ***p < 0.001.

Among the 144 DEGs, expression levels of six genes were found to associated with patient survival independently. Patients with lower expressions of BST2, CALB2, TNNT1, ENO2, HOXC6, and SYNGR3 had better cumulative survival than patients with higher expressions (Figure 5). Further, IHC of tissue samples suggested that in BRAF mutated colon samples, expression levels of BST2, CALB2, and ENO2 are significantly higher than those in BRAF wild-type tumor samples, as shown in Figures 6A–F, respectively.

### BRAF Mutation and Immunotherapy-Related Genes

**FIGURE 7:**
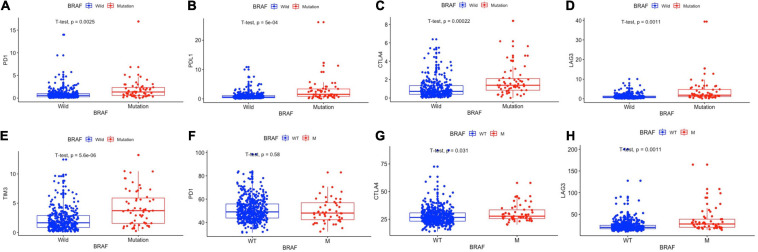
BRAF mutation was correlated with the expression levels of PD-1 **(A)**, PD-L1 **(B)**, CTLA-4 **(C)**, LAG-3 **(D)**, and TIM-3 **(E)** in the TCGA cohort and the expression levels of PD-1 **(F)**, CTLA-4 **(G)**, and LAG-3 **(H)** in the GEO cohort. Student’s *t*-tests were used for all analyses.

**FIGURE 8:**
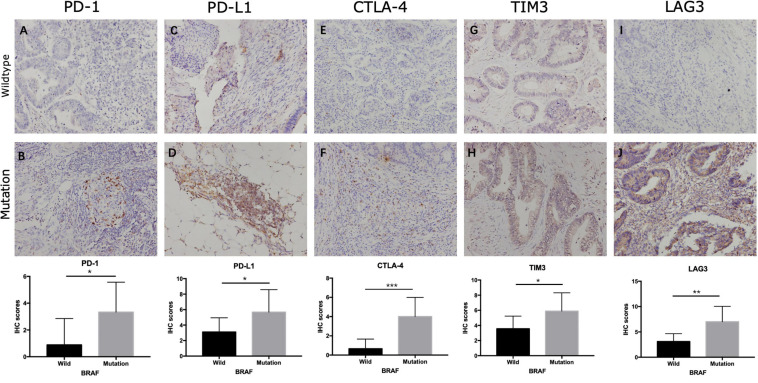
Representative IHC staining showing expression of immunotherapy-related genes in colon cancer. Staining of PD-1 **(A,B)**, PD-L1 **(C,D)**, CTLA-4 **(E,F)**, TIM-3 **(G,H)**, and LAG-3 **(I,J)** in BRAF wild-type **(A,C,E,G,I)** and BRAF mutated **(B,D,F,H,J)** samples at magnification ×200. *p < 0.05, **p < 0.01, ***p < 0.001.

Immunotherapies, especially the use of immune checkpoint inhibitors, comprise an emerging strategy for the treatment of colon cancer patients. Therefore, we further analyzed the relationship between BRAF mutation and target genes of immunotherapy. It is detected that patients with BRAF mutation in the TCGA cohort had higher expression of PD-1 (*p* = 0.0025), PD-L1 (*p* < 0.0001), CTLA-4 (*p* < 0.0001), LAG-3 (*p* = 0.0011), and TIM-3 (*p* < 0.0001) (Figures 7A–E). In patients from the GEO dataset, BRAF mutation colon cancer also had higher expressions of CTLA-4 (*p* = 0.031) and LAG-3 (*p* = 0.0011), but had no significant expression of PD-1 (*p* = 0.58) (Figures 7F–H). Immunochemistry of tumor tissues of patients suggest that the expression levels of PD-1 (Figures 8A,B), PD-L1 (Figures 8C,D), CTLA-4 (Figures 8E,F), TIM-3 (Figures 8G,H), and LAG-3 (Figures 8I,J) are significantly higher in BRAF mutated samples than in BRAF wild-type samples.

### BRAF Mutation Related Immune Cells

**FIGURE 9:**
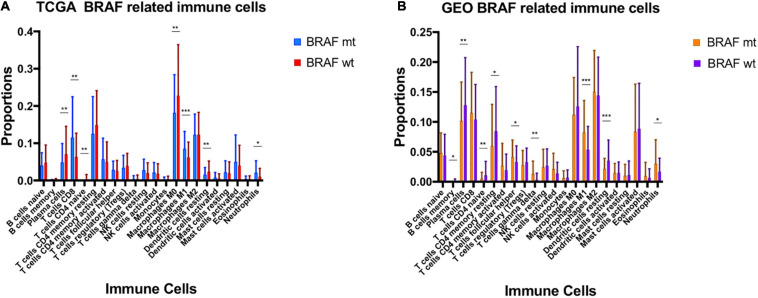
BRAF mutation was correlated with the proportion of immune cells in TCGA **(A)** and GEO **(B)**.

**FIGURE 10:**
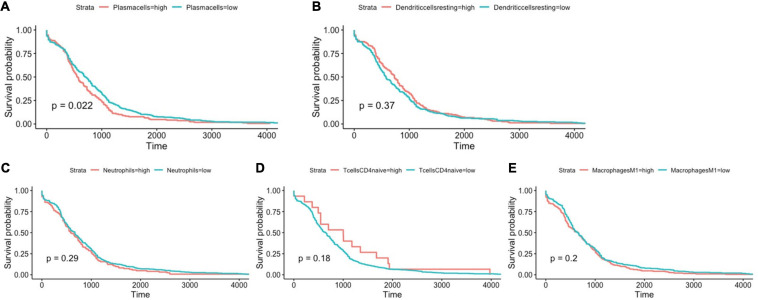
BRAF mutation-related immune cells, including plasma cells **(A)**, dendritic cells resting **(B)**, neutrophils **(C)**, T cells CD4 naïve **(D)**, and macrophages M1 **(E)** and their association with overall survival. Log-rank test for Kaplan–Meier curves was conducted to assess the association between overall survival and expression levels.

**FIGURE 11:**
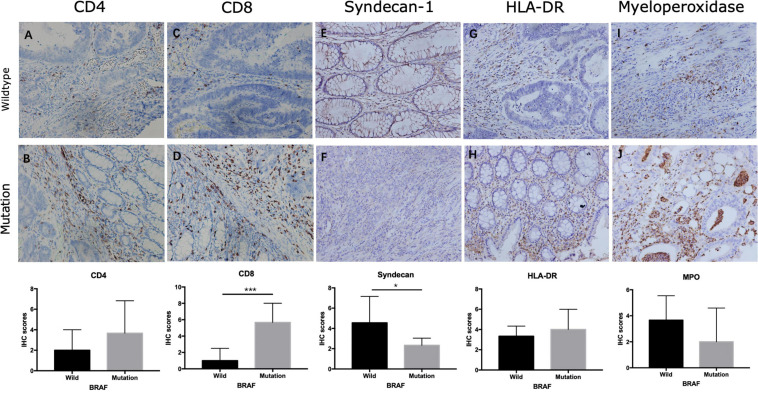
Expression of immune cells in colon cancer. Staining of CD4 **(A,B)**, CD8 **(C,D)**, Syndecan-1 **(E,F)**, HLA-DR **(G,H)**, and myeloperoxidase **(I,J)** in BRAF wild-type **(A,C,E,G,I)** and BRAF mutated **(B,D,F,H,J)** samples at magnification ×200. *p < 0.05, **p < 0.01, ***p < 0.001.

Using Cibersort algorithm, we identified the fraction of 22 immune cells in each tumor sample in the TCGA and GEO cohort. In TCGA samples, BRAF mutation was found to be significantly different in cell proportions of plasma cells, T cells CD8, T cells CD4 naïve, macrophages M0, macrophages M1, dendritic cells resting, and neutrophils (Figure 9A). In the GEO cohort, cell proportions of B cells memory, plasma cells, T cells CD4 naïve, T cells CD4 memory resting, T cells follicular helper, T cells gamma delta, macrophages M1, dendritic cells resting, and neutrophils were significantly different (Figure 9B). Therefore, the fractions of five immune cells, plasma cells, dendritic cells resting, neutrophils, macrophages M1, and T cells CD4 naïve were associated with BRAF mutation in both TCGA and GEO cohorts. Among these five immune cells, only the fraction of plasma cells was associated with patient survival (Figure 10) in the TCGA cohort. IHC staining proved that CD4 and CD8 cells are highly expressed in BRAF mutated samples, although the former did not illustrate statistical significance (Figures 11A–D). Interestingly, staining of Syndecan-1 (CD138), which is a cell surface heparan sulfate proteoglycan that could be used to detect plasma cells (Ajise et al., 2016), is highly expressed in BRAF wild-type tissues (Figures 11E,F). On the other hand, the staining intensity of HLA-DR, a common marker for macrophage M1 (Jayasingam et al., 2020), and myeloperoxidase (MPO), a biomarker for infiltrating neutrophils (Mariani and Roncucci, 2017), did not show significant difference between BRAF wild-type and BRAF mutated tissues (Figures 11G–J).

### MSI Status in BRAF Mutated Colon Cancer

**FIGURE 12:**
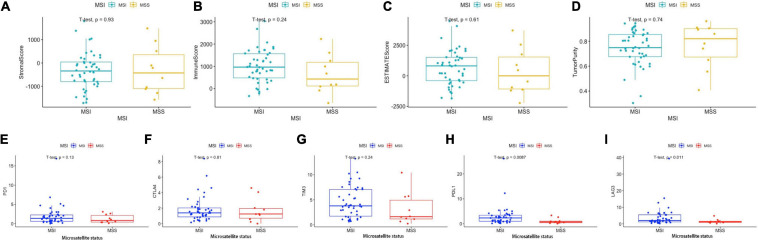
Immune scores and immunotherapy-related genes according to MSI status. Stromal score **(A)**, immune score **(B)**, ESTIMATE score **(C)**, and tumor purity **(D)** in MSI and MSS groups. Expression levels of PD-1 **(E)**, CTLA-4 **(F)**, LAG-3 **(G)**, PD-L1 **(H)**, and TIM-3 **(I)** in the MSI and MSS groups. Student’s *t*-tests were used for all analyses.

As mentioned above, BRAF mutated colon cancer is associated with higher MSI. We then divided the 59 BRAF mutated patients into microsatellite stable (MSS) subgroup and microsatellite instable (MSI) subgroup. We found that stromal score, immune score, ESTIMATE score, and tumor purity did not differ in these two groups (Figures 12A–D). PD-1 expression, CTLA expression, and TIM-3 expression did not illustrate significant difference in these subgroups either (Figures 12E–H). However, the MSI group showed significantly higher expression of PD-L1 (*p* = 0.009) (Figure 12H) and LAG-3 (*p* = 0.011) (Figure 12I) than the MSS group.

## Discussion

BRAF mutation is one of the most important mutation subtypes in colon cancer. Its unique tumorigenesis process suggests a different biological and pathological landscape from wild-type colon cancer. BRAF mutation is often associated with MSI, minimal chromosomal instability, and high rate of recurrence (Taieb et al., 2019). The presence of BRAF mutation is also correlated with CpG island methylator phenotype (CIMP), which can lead to the inactivation of the MLH1 promoter, resulting in an MMR deficiency. In the present study, we have identified immune characteristics of BRAF mutated colon cancer and have investigated the immune microenvironment of BRAF mutated colon tumors.

Through assessment of immune scores, we identified that BRAF mutated colon cancer had more stromal cells, more immune cell infiltration, and lower tumor purity in tumor tissue. Researches have suggested that stromal cells are often recruited by tumor cells from nearby stroma and play a critical role in tumor angiogenesis, proliferation, invasion, metastasis, and drug resistance (Bussard et al., 2016; Denton et al., 2018). The stromal cells could also transform into tumor-associated stromal cells, which would further secrete many cytokines to promote tumorigenesis such as IL-6, IL-8, and vascular endothelial growth factor (Bussard et al., 2016). Galland and Stamenkovic (2020) have indicated that stromal cells may have dual effects on tumor progression; that is, as stromal cells compromise several subgroups of cell populations, they could promote and constrain tumor growth at the same time. Reeducating and targeting stromal cells may both serve as effective strategy for antitumor therapies (Quail and Joyce, 2013). We have also found that BRAF mutation is correlated with higher proportions of neutrophils and macrophages M1, and lower proportions of plasma cells, dendritic cells resting, and T cells CD4 naïve. BRAF V600E mutation in colon cancer is often associated with consensus molecular subtype 1 (CSM1), which correlates with considerable immune infiltrations and activation of immune response pathways, especially with larger populations of type 1 T helper cells, cytotoxic T cells, and NK cells (Dienstmann et al., 2017). However, roles of these immune cells in the process of oncogenesis and their responses to chemotherapy, targeted therapy, and immunotherapy need further investigation.

Our pathway enrichment analysis reveals that cytokine-related pathways, such as chemokine signaling, cytokine receptor interaction, viral protein interaction with cytokine, and cytokine receptors, are differently expressed between BRAF mutated and BRAF wild-type tumors. The result is consistent with previous studies that BRAF mutant MSI-H colon cancer is associated with the overexpression of stromal cell-derived factor-1 (SDF-1; also called CXCL12) and chemokine (C-X-C motif) receptor 4 (CXCR4), thereby suggesting chemokines like CXCR4 may serve as future therapeutic targets (Molina-Cerrillo et al., 2020). Also, it is found that proangiogenic chemokines are highly expressed in BRAF mutated cell lines of colon cancer (Khan et al., 2014). Therefore, anti-VEGF therapies are recommended for BRAF mutated cancer patients. TRIBE study has proven the efficacy of FOLFOXIRI + bevacizumab as well for this subgroup of colon cancer patients (Cremolini et al., 2015). In addition to FOLFOXIRI chemotherapy, BRAF and MEK inhibitors are being tested in clinical trials to treat BRAF mutated colon cancer (Wu, 2018). The most recent therapy recommended for patients with BRAF mutant metastatic colon cancer is the binimetinib, encorafenib, and cetuximab triplet therapy from the BEACON Study (Kopetz et al., 2019).

Among the 144 DEGs between BRAF mutation and BRAF wild-type subtypes, BST2, CALB2, TNNT1, ENO2, HOXC6, and SYNGR3 were associated with overall survival in the TCGA dataset. Similar results were found that elevated BST2 level, HOXC6, and TNNT1 level are correlated with poor prognosis in colon cancer patients, while TNNT1 protein may be mediated through the process of epithelial–mesenchymal transition (Chiang et al., 2015; Hao et al., 2020; Yuan et al., 2020). CALB2 is a calcium binding protein from the EF hand family and is expressed in the majority of poorly differentiated colon cancer (Häner et al., 2010). It is also found that CALB2 may serve as a mediator for cell apoptosis in 5-FU-treated colon cancer through the mitochondrial pathway (Stevenson et al., 2011).

Interestingly, we also found that BRAF mutation was associated with overexpression in many immunotherapeutic targets, such as PD-1, PD-L1, CTLA-4, LAG-3, and TIM-3. Results from the IHC further prove that tumors with BRAF mutation have higher expression of these immunotherapy-related proteins. The Food and Drug Administration has already approved the clinical use of PD-1 inhibitors pembrolizumab and nivolumab for MSI-high tumors. Although the earlier Checkmate-142 study suggested that monotherapy of nivolumab has achieved an ORR of 25% in BRAF mutant tumors and 41% in KRAS/BRAF wild-type tumors, the most recent result has proven that nivolumab plus low-dose ipilimumab has achieved satisfying outcome for MSI-H/dMMR metastatic colorectal cancer (Overman et al., 2017; Lenz et al., 2020). The combination of two immunotherapies achieved an ORR of 71% in BRAF mutant tumors and an ORR of 60% in all patients (Lenz et al., 2020). In the phase II KEYNOTE-164 trial, ORRs of 20% and 55% were observed for second or further line treatment and third or further line treatment, respectively, for pembrolizumab-treated patients with BRAF mutant tumors who were resistant to chemotherapy (Le et al., 2020). This result demonstrated that combination immunotherapies had robust clinical benefit for colon cancer patients with high MSI, especially those with BRAF mutations. Since around 95% of metastatic colorectal cancer patients have MSS-type tumors, which act limited to single-agent immunotherapy, it is even inspiring that Japanese scientists reported an antitumor benefit from the combination of regorafenib plus nivolumab for advanced colorectal cancer patients in the phase Ib REGONIVO trial (Fukuoka et al., 2020).

Notably, although it is well acknowledged that dissimilar treatment strategies should be applied to MSS-type BRAF mutated colon cancer and MSI-type BRAF mutated colon cancer, no study has investigated tumor microenvironment among these subgroups yet. A review from Gelsomino et al. (2016) suggested that although patients with MSI-H generally had better prognosis than patients with MSS status, this favorable effect seemed to be partially mitigated by BRAF mutation. It should also be noted that the expression pattern of BRAF mutant colorectal cancer is very diverse, and several subtypes have been identified. Even two categories of BRAF V600E mutation have been identified with distinct molecular pattern, with one displaying high EMT activation and immune infiltration and the other one showing dysregulation in cell cycle checkpoints (Barras et al., 2017). Therefore, further researches may look into the precise targeted therapies to these subpopulations.

Recently, a follow-up study from the Checkmate-142 trial suggested that for patients who simultaneously had BRAF mutation and MSI status, nivolumab (NIVO) + low-dose ipilimumab (IPI) as first-line therapy received a remarkable ORR of 71% (12/17) (Lenz et al., 2020). Taking into account that BRAF mutated colon cancer tends to have higher expression levels of immunotherapy-related genes, such as PD-1, PD-L1, CTLA-4, and LAG-3, the use of checkpoint inhibitors in the Checkmate-142 trial was favorable and indicated that BRAF mutated colon cancer might present with an immunosuppressive microenvironment, which was in agreement with our conclusion.

A limitation of our study was that the patients involved in our study, including patients from the TCGA dataset, the GEO dataset, and our hospital, are all postoperative ones. Hence, the patients with late-stage diseases were not included for analyses. In addition, information on postoperative treatments was not available. Further validation of the treatment efficacy in patients may provide more determined conclusions.

In conclusion, the present study provides insights into the tumor microenvironment in BRAF mutated colon cancer and discussed potential therapeutic targets from the perspective of immune biology. Immunotherapies may become fundamental treatments for BRAF mutated colon cancer in the future.

## Data Availability Statement

The datasets presented in this study can be found in online repositories. The names of the repository/repositories and accession number(s) can be found in the article/supplementary material.

## Ethics Statement

The studies involving human participants were reviewed and approved by Research Ethics Committee of Beijing Friendship Hospital. Written informed consent for participation was not required for this study in accordance with the national legislation and the institutional requirements.

## Author Contributions

ZL and ZB contributed to the conceptualization. SC and KL contributed to the investigation. SC, YZ, JS, CJ, and JG contributed to the analysis. SC, ZL, and KL contributed to the writing – original draft. ZL, ZB, and HP contributed to the writing – review and editing, and final approval. All authors contributed to the article and approved the submitted version.

## Conflict of Interest

The authors declare that the research was conducted in the absence of any commercial or financial relationships that could be construed as a potential conflict of interest.

## Publisher’s Note

All claims expressed in this article are solely those of the authors and do not necessarily represent those of their affiliated organizations, or those of the publisher, the editors and the reviewers. Any product that may be evaluated in this article, or claim that may be made by its manufacturer, is not guaranteed or endorsed by the publisher.

## Footnotes

1 https://tcga-data.nci.nih.gov/tcga/

2 https://github.com/CJ-Chen/TBtools

3 https://github.com/therneau/survival

4 http://www.sthda.com/english/rpkgs/survminer/

5 http://timer.cistrome.org/

6 https://cibersort.stanford.edu/
